# Attenuation of Diabetic Nephropathy in Diabetic Mice by Fasudil through Regulation of Macrophage Polarization

**DOI:** 10.1155/2020/4126913

**Published:** 2020-06-30

**Authors:** Fajiang Xie, Jiesen Lei, Maoxia Ran, Yan Li, Li Deng, Jian Feng, Yi Zhong, Jiafu Li

**Affiliations:** ^1^Collaborative Innovation Center for Prevention and Treatment of Cardiovascular Disease of Sichuan Province, Department of Cardiology, The Affiliated Hospital of Southwest Medical University, Luzhou, Sichuan, China; ^2^Department of Cardiology, Dazhou Central Hospital, Dazhou, Sichuan, China; ^3^Department of Rheumatology, The Affiliated Hospital of Southwest Medical University, Luzhou, Sichuan, China

## Abstract

Inflammation and fibrosis induced by hyperglycemia are considered to play a critical role in the pathogenesis of diabetic nephropathy. As macrophage polarization may determine the severity and progression of inflammation, regulation of macrophage polarization may be an effective method to treat diabetic complications. Fasudil, a potent Rho-kinase inhibitor, reportedly exhibits anti-inflammatory activity. However, whether fasudil reduces hyperglycemia-induced diabetic nephropathy via regulation of macrophage polarization remains unclear. In this study, we investigate the effect of fasudil on diabetic nephropathy in streptozotocin-induced type 1 diabetic mice. Our data showed that fasudil significantly decreased urinary protein and serum creatinine in diabetic mice, whereas it had no effect on the body weight and blood glucose. We also found increased M1-type macrophages and related proinflammatory cytokines, adverse fibrosis in renal tissue of diabetic mice. Interestingly, treatment of diabetic mice with fasudil increased the number of M2-type macrophages and related anti-inflammatory cytokines, which attenuated renal injury in diabetic mice. Taken together, the results of this study suggest that fasudil could slow the progression of diabetic nephropathy. The possible mechanism might be associated with its induction of M2 macrophage polarization and the reduction of M1 macrophage polarization and inflammation.

## 1. Introduction

Diabetic nephropathy (DN) is the most common microvascular complication of diabetes mellitus and are also the most common etiology of end-stage renal disease [1, 2]. Exudation of inflammatory cells and overexpression of proinflammatory cytokines are important pathogeneses of DN. Long-term activation of inflammation can result in renal fibrosis and remodeling [3]. Regulation of inflammatory responses is an important method for DN treatment.

Macrophages are key cells to initiate inflammation. An increase in macrophage exudation can be observed in kidney tissues in early DN while still having normal kidney functions [4]. The number of interstitial macrophages is closely associated with proteinuria, glomerular destruction, and kidney function [4–6]. Influenced by the local microenvironment, macrophages differentiate into at least two subtypes to participate in inflammatory responses. This process is called macrophage polarization and primarily produces classically activated macrophages (M1 type) and alternatively activated macrophages (M2 type). M1 macrophages upregulate the expression of inducible nitric oxide synthase (iNOS) and inflammatory cytokines, and excessive polarization can lead to tissue damage. M2 macrophages upregulate the expression of arginase-1 (Arg-1) and anti-inflammatory cytokines to play an anti-inflammatory effect, which is conducive to tissue repair [7]. The number of M1 macrophages in DN significantly increases, and the number of M2 macrophages significantly decreases [8]. By examining the M1 (CD 80 and CD86) and M2 makers (CD163 and 206), Lu et al. [8] have found that compared to nondiabetic rats, M1 macrophages were dramatically increased in streptozotocin- (STZ-) induced DN rats while the levels of M2 macrophages were reduced, suggesting the M1/M2 ratio imbalance is involved in the mechanisms of DN. Recently, a study by Guo et al. [9] confirmed the previous finding by measuring the markers of M1 and M2 macrophages in high glucose condition. After stimulation with high glucose, macrophages increased the expression of M1 macrophage marker and decreased the expression of M2 macrophage marker compared with those exposed to normal glucose. Moreover, multiple lines of evidence also have demonstrated that inhibiting M1 macrophages and enhancing M2 macrophages with various treatments can prevent streptozotocin-induced kidney injury [8, 10, 11]. Therefore, these findings indicate that regulation of macrophage polarization and reversal of the M1/M2 ratio may be improve DN.

The Rho-associated coiled-coil containing protein kinase (ROCK) signaling pathway regulates cell behaviors including cell proliferation, migration, and apoptosis to play a molecular switch role [12]. Fasudil can specifically bind to the ATP-dependent kinase domain in ROCK to inhibit its activity. Fasudil is currently the only clinically approved ROCK inhibitor. Because of its powerful vasodilation function, fasudil has been extensively applied in vasospastic diseases, such as subarachnoid hemorrhage and ischemic heart disease [13]. Studies have shown that fasudil can treat experimental autoimmune encephalomyelitis in mice [14, 15]. One of its potential mechanisms is to induce M2 polarization of macrophages and inhibit M1 polarization to block inflammatory responses [14–16]. Although it has been reported that fasudil can inhibit renal interstitial fibrosis induced by unilateral ureteral obstruction, there is still no report on whether fasudil can regulate macrophage polarization to attenuate renal fibrosis induced by hyperglycemia [17]. Therefore, this study used fasudil intervention in the STZ-induced type 1 diabetic mouse model to observe macrophage polarization and renal fibrosis.

## 2. Materials and Methods

### 2.1. Reagents

STZ was purchased from Sigma (USA). Fasudil hydrochloride injection was purchased from Tianjin Chase Sun Pharmaceutical Co., Ltd. (China). CD68, CD11c, and CD206 antibodies were all purchased from the ProteinTech Group (USA). TNF-*α*, IL-6, and IL-10 antibodies were all purchased from the Bioworld Technology (USA). Phosphorylated myosin phosphatase target subunit 1 (p-MYPT1), iNOS, and Arg-1 antibodies were all purchased from CST (USA). *β*-Actin antibodies were purchased from Abcam (UK). The BCA protein concentration determination reagent kit was purchased from Nanjing KeyGen Biotech Co., Ltd. (China).

### 2.2. Animals and Treatment

A total of 60 SPF-grade and healthy male C57BL/6 mice aged 6-8 weeks with a body weight of 19-21 g were purchased from Chengdu Dossy Experimental Animals Co., Ltd. (China). During the experiment, the animals could access water and common feed *ad libitum*. The room temperature was 23 ± 1°C, and a 12 h/12 h light/dark cycle was maintained. The experimental protocol was approved by the Animal Care and Use Committee of the Southwest Medical University (Luzhou, China). And all animal experiments were performed according to the National Institutes of Health guide for the care and use of laboratory animals (NIH Publications No. 8023, revised 1978).

After adaptive feeding for 1 week, the animals were randomly divided into the following groups: the control group (Ctrl), fasudil group (FD), diabetes mellitus group (DM), and DM+different doses of fasudil groups. In our previous study, we have found that fasudil at 10 mg/kg, 40 mg/kg, and 60 mg/kg improved myocardial damage in diabetic C57BL/6 mice [18]. Therefore, in this study, we divided fasudil intervention group into DM+10 mg/kg fasudil group (DM+LFD), DM+40 mg/kg fasudil group (DM+MFD), and DM+60 mg/kg fasudil group (DM+HFD). Each group had 10 animals. After fasting for 12 h, the mice in the DM group and the treatment groups received intraperitoneal injections of 1% STZ at 80 mg/kg, while mice in the Ctrl and FD groups received intraperitoneal injections of an equal volume of 0.1 mmol/L sodium citrate buffer daily for 5 d. After 72 h, blood samples were obtained from the tail. A blood glucose value of greater than 16.7 mmol/L was considered successful model establishment. After model establishment, fasudil intervention groups received intraperitoneal injection of 10 mg/kg, 40 mg/kg, and 60 mg/kg fasudil, respectively, once a day; the FD group received fasudil (40 mg/kg), and the Ctrl and DM groups received an intraperitoneal injection of an equal volume of normal saline daily for 12 weeks. The 24 h urine from the mice was collected in metabolic cages to quantify urinary protein. Blood samples were collected for determination of creatinine. Mice were sacrificed, and the kidneys were fixed in 4% paraformaldehyde to prepare paraffin sections at a thickness of 4 *μ*m. Some kidney tissues were frozen in a -80°C freezer for western blot detection.

### 2.3. Biochemical Analysis

24-hour urine was collected in a metabolic cage and the urine volume should be no less than 1 mL. The blood sample was collected and centrifuged to ensure that the serum level was no less than 500 *μ*L. Quantitation of 24 h urine and serum creatinine (Scr) were measured by enzymatic assay or colorimetry using an automatic biochemical analyzer (ADVIA 2400, Siemens, Erlangen, Germany) as described previously [19].

### 2.4. Renal Histology Analysis

Paraffin sections from each group were selected for conventional deparaffinization and the H&E and Masson staining. The results were observed and photographed under a light microscope (400×) (BA400Digital, Motic Group). The Masson staining was used to assess the collagen fibers and the ratio of fibrotic lesions to the examined area. The Masson stainings were examined randomly, and the Image-Pro Plus 6.0 analysis system was used for the quantitative evaluation of The Masson staining-positive tissue percent area as described previously [20].

### 2.5. Immunohistochemistry Analysis

After deparaffinization, sections from each group were immersed in 0.01 mmol/L sodium citrate buffer (pH 6.0) and heated in a microwave oven until boiling. The power was turned off, and the treatment was repeated again after 5 min. After cooling, sections were washed with PBS for 5 min twice for antigen retrieval. Goat serum was used to block the sections for 20 min at room temperature. CD11c (1 : 50), CD206 (1 : 100), IL-6 (1 : 100), TNF-*α* (1 : 100), and IL-10 (1 : 50) antibodies were then added and incubated at 4°C overnight. The secondary antibody was then added and incubated at 37°C for 30 min. The sections were washed with PBS for 5 min three times, and the results were developed using DAB. Finally, the sections were counterstained with hematoxylin, dehydrated, and mounted in neutral balsam. The results were observed and photographed under a light microscope (OLYMPUS BX53, Japan). Five different observation areas were selected from the staining sections of IL-6, TNF-*α*, and IL-10 under the 100-fold microscope, and the photographs of these areas were taken by a microscope under 400-fold microscope. The integrated optical density (IOD), mean density (MD), and area (Area) of all five field images were measured by the Image-Pro Plus 6.0 analysis system. Firstly, an observation area was selected from the staining section, followed by the measuring of the IOD and Area of the selected observation area by the analysis system. And then, the MD was calculated as IOD/Area. Finally, the MD of five field images were used for statistical analysis. Moreover, the number of CD11c and CD206 positive cells was calculated in the same way.

### 2.6. Western Blot

Mouse kidney tissues were thawed in a 37°C water bath, and RIPA lysis buffer was added at 1 : 10 (weight:volume). The tissues were rapidly minced and lysed on ice for 10 min. The lysis solution was collected and centrifuged at 4°C and 12000 r/min for 10 min. The supernatant was collected, and the protein concentration was measured according to the instructions of the BCA protein quantitation reagent kit. According to the required sample volume, 5× loading buffer was added at a 4 : 1 ratio. After mixing thoroughly, the proteins were denatured at 95°C for 15 min in a thermal cycler. Resolving gels (8% and 10%) and stacking gels (5%) were prepared, and 60 *μ*g of protein was loaded and separated using electrophoresis. The protein sample was transferred onto a PVDF membrane and blocked in 5% BSA solution for 2 h. Primary antibodies, p-MYTP1 (1 : 500), iNOS (1 : 200), Arg-1 (1 : 300), CD68 (1 : 200), and *β*-actin (1 : 5000), were added and incubated at 4°C overnight. The membrane was washed with TBST (pH 7.4) for 5 min three times and then incubated with the corresponding secondary antibodies (1 : 5000) at room temperature for 2-3 h. The membrane was washed with TBST for 10 min three times, and the results were developed using ECL chemiluminescence solution. The analysis of protein bands was performed using the ChemiDoc XRS+ Imaging System with Image Lab Software (Bio-Rad).

### 2.7. Statistical Analysis

Statistical analyses were performed using the SPSS 17.0 statistical software. Data are expressed as the mean ± SD. The comparison of the mean values among multiple samples was performed using one-way analysis of variance (ANOVA) followed by the Bonferroni post hoc test. A value of *P* < 0.05 was considered statistically significant.

## 3. Results

### 3.1. Fasudil Reduced Urinary Protein and Scr in Diabetic Mice

**Figure 1:**
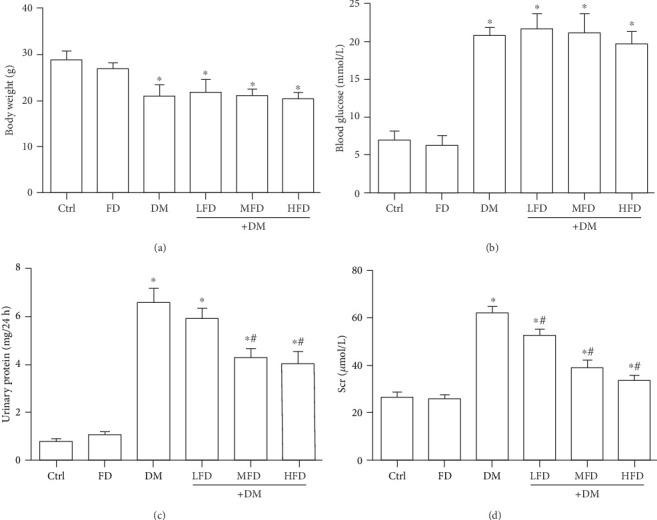
Fasudil reduced urinary protein and Scr in diabetic mice. (a) Body weights of mice from different treatments. (b) Blood glucose of mice from different treatments. (c) Urinary protein of mice from different treatments. (d) Scr of mice from different treatments. The data are presented as the mean ± SD (*n* = 10). ∗*P* < 0.05 vs. the Ctrl group; ^#^*P* < 0.05 vs. the DM group.

As shown in Figure 1, compared to that of the Ctrl group, the body weight of the mice in DM group decreased significantly (*P* < 0.05), and the blood glucose, urinary protein, and Scr increased significantly (*P* < 0.05). Compared to those in the DM group, differences in body weight and blood glucose among all treatment groups were not statistically significant (Figures 1(a) and 1(b)). The increased urinary protein excretion in the DM group was attenuated by treatment in the DM+MFD or the DM+HFD group (*P* < 0.05) (Figure 1(c)). There was no significant difference between the DM+LFD group and the DM groups. Compared with the DM group, DM+LFD, DM+MFD, or DM+HFD group had a decreased Scr (*P* < 0.05) (Figure 1(d)). The differences in all indicators between FD group and Ctrl group were not statistically significant.

### 3.2. The Pathological Changes of Renal Tissues in Each Group

**Figure 2:**
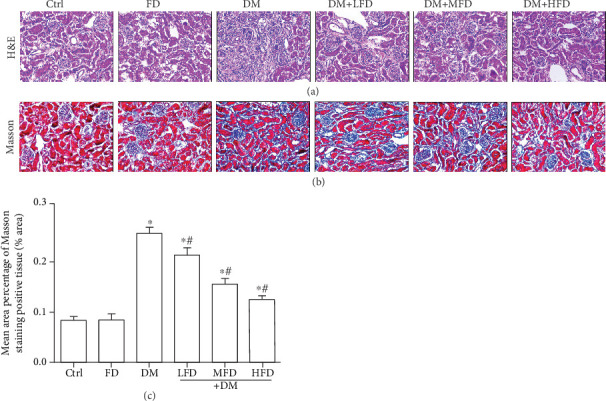
The pathological changes of renal tissues in each group. (a) Representative images of H&E staining for each group, light microscopy (400×). (b) Representative images of the Masson staining for each group, light microscopy (400×). (c) Mean area percentage of the Masson staining-positive tissue in renal tissue specimens. Data are expressed as mean ± SD, ∗*P* < 0.05 vs. the Ctrl group; ^#^*P* < 0.05 vs. the DM group.

Observation using the H&E and Masson staining showed that the arrangement of glomerular, tubular, and mesangial structures was ordered in the Ctrl and FD groups, while the clear tissue outline and a small amount of collagen fibers in the interstitium were also observed in the Ctrl and FD groups (Figure 2). In the DM group, there were glomerular hypertrophy, different levels of degeneration and necrosis of renal tubular epithelial cells, an increased mesangial matrix, a disordered cell arrangement, and a large amount of collagen fibers in the interstitium. A quantitative analysis showed that there was a significant increase in the mean area percentage of the Masson staining-positive tissue in the DM group relative to the Ctrl group (*P* < 0.05, Figure 2(c)). Moreover, treatment of diabetic mice with fasudil at various doses resulted in a decrease in the mean area percentage of the Masson staining-positive tissue in a dose-dependent manner (Figure 2(c)).

### 3.3. Fasudil Inhibited M1 Macrophage Polarization and Proinflammatory Cytokines in Renal Tissues of Diabetic Mice

**Figure 3:**
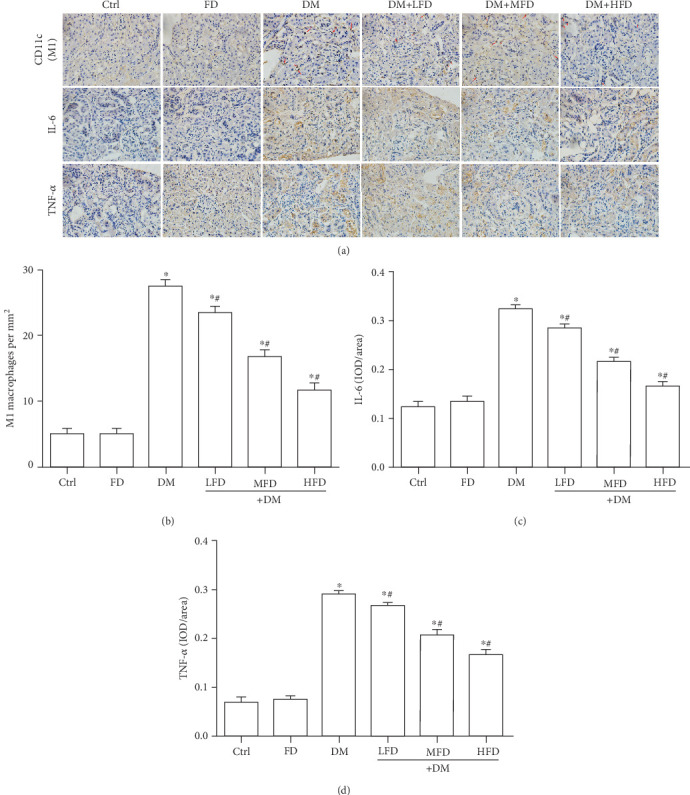
Fasudil inhibited M1 macrophage polarization and proinflammatory cytokines in renal tissues of diabetic mice. (a) Representative immunohistochemical staining of CD11c (a marker for M1 macrophages; red arrows), IL-6, and TNF-*α* in renal tissues. (b) Quantitative analysis for the number of M1 macrophages in renal tissue specimens. Data are expressed as mean ± SD, ∗*P* < 0.05 vs. the Ctrl group; ^#^*P* < 0.05 vs. the DM group. (c) Quantitative analysis of the protein expression of IL-6 in renal tissue specimens. Data are expressed as mean ± SD, ∗*P* < 0.05 vs. the Ctrl group; ^#^*P* < 0.05 vs. DM group. (d) Quantitative analysis of the protein expression of TNF-*α* in renal tissue specimens. Data are expressed as mean ± SD, ∗*P* < 0.05 vs. the Ctrl group; ^#^*P* < 0.05 vs. the DM group.

Representative images of renal sections from different group stained with CD11c, an M1 macrophage marker, IL-6, and TNF-*α* are shown in Figure 3(a). The quantitative analysis showed that there was a significant upregulation of M1 macrophages, IL-6, and TNF-*α* in the DM group relative to the Ctrl group (*P* < 0.05, Figures 3(b)–3(d)). Moreover, treatment of diabetic mice with fasudil at various doses resulted in a descending number of M1 macrophages in a dose-dependent manner (Figure 3(b)), along with decreased protein expression of IL-6 and TNF-*α* (Figures 3(c) and 3(d)).

### 3.4. Fasudil Increased M2 Macrophage Polarization and Anti-Inflammatory Cytokines in Renal Tissues of Diabetic Mice

**Figure 4:**
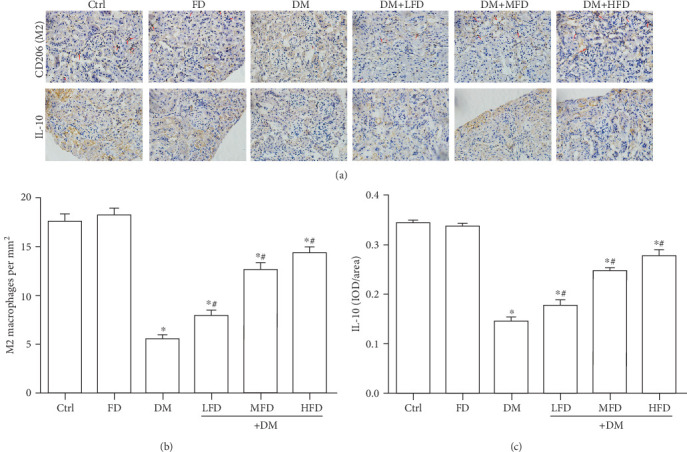
Fasudil increased M2 macrophage polarization and anti-inflammatory cytokines in renal tissues of diabetic mice. (a) Representative immunohistochemical staining of CD206 (a marker for M2 macrophages; red arrows) and IL-10 in renal tissues. (b) Quantitative analysis for the number of M2 macrophages in renal tissue specimens. Data are expressed as mean ± SD, ∗*P* < 0.05 vs. the Ctrl group; ^#^*P* < 0.05 vs. the DM group. (c) Quantitative analysis of the protein expression of IL-10 in renal tissue specimens. Data are expressed as mean ± SD, ∗*P* < 0.05 vs. the Ctrl group; ^#^*P* < 0.05 vs. the DM group.

Representative images of renal sections from different groups stained with CD206, an M2 macrophage marker, and IL-10 are shown in Figure 4(a). The quantitative analysis showed that there was a significant downregulation of M2 macrophages and IL-10 in the DM group relative to the Ctrl group (*P* < 0.05, Figures 4(b) and 4(c)). Moreover, treatment of diabetic mice with fasudil at various doses resulted in ascending number of M2 macrophages in a dose-dependent manner (Figure 4(b)), along with increased protein expression of IL-10 (Figure 4(c)).

### 3.5. Correlation between ROCK Activity and Macrophage Polarization

**Figure 5:**
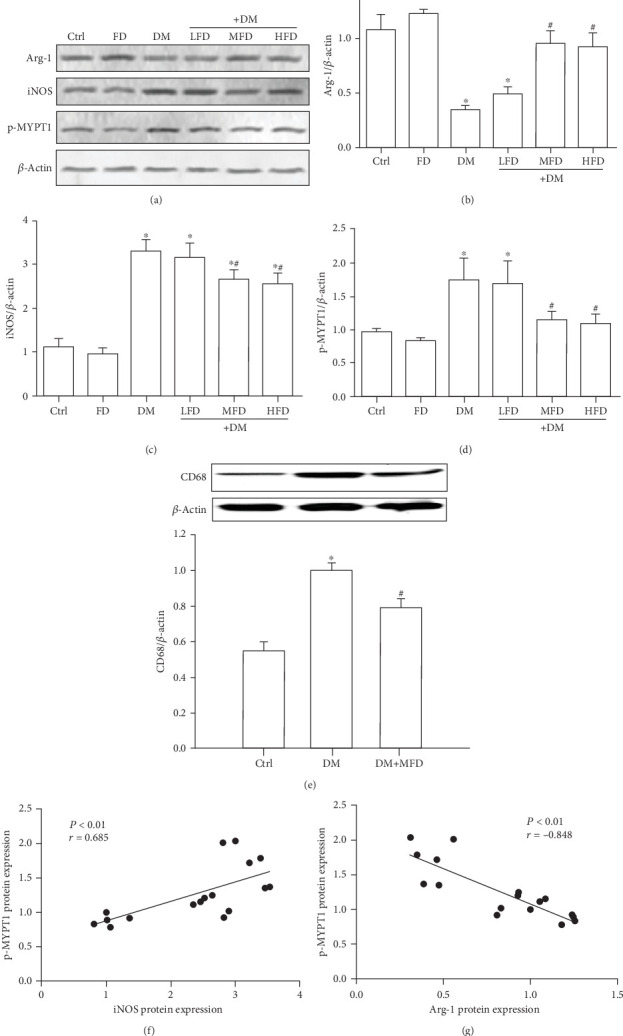
Correlation between ROCK activity and macrophage polarization. (a) Western blot analysis of Arg-1, iNOS, p-MYTP1, and *β*-actin expression in renal tissue specimens. Data are expressed as mean ± SD of three independent experiments. (b–d) Quantification of Arg-1, iNOS, and p-MYTP1. The data are presented as the mean ± SD.∗*P* < 0.05 vs. the Ctrl group; ^#^*P* < 0.05 vs. the DM group. (e) Western blot analysis of CD68 and *β*-actin expression in renal tissue specimens. Data are expressed as the mean ± SD of three independent experiments. (f) Correlation between p-MYTP1 and iNOS (*r* = 0.685, *P* < 0.01). (g) Correlation between p-MYTP1 and Arg-1 (*r* = −0.848, *P* < 0.01).

Representative western blot analyses of Arg-1 (an M2-specific maker), iNOS (an M1-specific maker), and p-MYPT1 are shown in Figure 5(a). Compared to that in the Ctrl group, the protein level of Arg-1 decreased in the DM group (*P* < 0.05, Figure 5(b)). Compared to the DM group, the protein level of Arg-1 increased in the DM+MFD and DM+HFD groups (*P* < 0.05, Figure 5(b)). The protein level of iNOS in the DM group increased relative to the Ctrl group (*P* < 0.05, Figure 5(c)). Compared to the DM group, the protein level of iNOS in the DM+MFD and DM+HFD groups decreased (*P* < 0.05, Figure 5(c)). MYPT1 is one of the downstream binding substrates of ROCK. The level of p-MYPT1 can effectively reflect ROCK activity [21]. Compared to that in the Ctrl group, the protein level of p-MYPT1 in the DM group increased (*P* < 0.05, Figure 5(d)). Compared to the DM group, the protein levels of p-MYTP1 in the DM+MFD and DM+HFD groups decreased (*P* < 0.05, Figure 5(d)). As shown in Figure 5(e), compared with the control, DN mice increased the total macrophage numbers. Treatment DN mice with fasudil alleviated the total macrophage numbers compared with the DM group, as illustrated by the protein expression of CD68 (a macrophage maker). Furthermore, the p-MYPT1 protein level was positively correlated with the iNOS protein level (*r* = 0.685, *P* < 0.01) (Figure 5(f)) and was negatively correlated with the Arg-1 protein level (*r* = −0.848, *P* < 0.01) (Figure 5(g)). The above data indicate that the ROCK signaling pathway correlated with macrophage polarization in renal tissues of diabetic mice.

## 4. Discussion

The early pathological changes of DN are mainly glomerulosclerosis, renal arteriolar damage, and renal tubular degeneration; interstitial fibrosis is a prominent presentation in the late stage [22]. In this study, a type 1 diabetes mellitus mouse model was established through intraperitoneal injection of STZ. The observation results showed that the kidneys of diabetic mice had an increase in urinary proteins and a reduction in kidney function.

Under pathological conditions, monocytes migrate and exude from the circulatory system to the tissue and differentiate into macrophages. Undifferentiated M0 macrophages are induced in different microenvironments to differentiate into different types. M1 macrophages are mainly induced by lipopolysaccharides (LPS) and interferon-*γ* (IFN-*γ*). In addition, proinflammatory cytokines, such as TNF-*α* and IL-12, also have induction functions. Activated M1 macrophages secrete a large amount of reactive nitrogen to enhance Th1 cell immunity. The release of a large amount of inflammatory mediators has powerful antibacterial and cytotoxic functions that produce anti-infection and antitumor effects. These mediators are first exuded at the initial stage of acute injury inflammation to play a role in the necrotic tissue clearance [10]. M2 macrophages are induced by different factors to differentiate into M2a, M2b, and M2c subtypes that have different functions. The M2a type is induced by IL-4 and IL-13, the M2b type is induced by immune complexes and IL-1, and the M2c type is induced by IL-10, transforming growth factor-*β* (TGF-*β*), and glucocorticoids. The M2a type can inhibit the release of inflammatory mediators, such as INF-*γ*, IL-1, and IL-6, and can promote TGF-*β* expression and intercellular matrix deposition. However, its phagocytic function is poor. Because it has tissue repair function, it is also referred to as the “tissue repair macrophage” [23]. The M2b and M2c types limit tissue destruction caused by abnormal sustained activation of M1 macrophages by mediating chronic inflammation and do not directly participate in tissue repair; therefore, they are referred to as “regulatory macrophages” [24]. M1 and M2 macrophages have different functions at different stages of inflammation. The dynamic changes in the ratio of M1/M2 macrophages determine the progression and severity of inflammation. The study by Cucak et al. [25] showed that the level of the M1 macrophage marker protein CD11c decreased and the level of the M2 macrophage marker protein galectin-3 increased in kidney tissues in the db/db mouse DN model and that this phenomenon could be reversed by enalapril treatment. Therefore, these authors considered that DN mainly had M2 macrophage polarization. Interestingly, increasing evidence has indicated that the number of M1 macrophages increases and the number of M2 macrophages decreases in kidney tissues during DN. The M1/M2 ratio imbalance is one pathogenesis of DN, and regulation of macrophage polarization directly or indirectly may be a strategy for DN treatment [26–28].

In this study, we showed that M1 macrophage polarization and proinflammatory cytokine expression increased, M2 macrophage polarization and anti-inflammatory cytokine expression decreased in DN mice. When treating the DN mice with fasudil, we found that M1 macrophage polarization and proinflammatory cytokine expression reduced, and M2 macrophage polarization and anti-inflammatory cytokine expression increased. These results suggest that fasudil regulated macrophage polarization to reverse the abnormal M1/M2 macrophage ratio in DN. Previous studies have demonstrated the ability of fasudil to regulate macrophage polarization *in vivo* and *in vitro*. In experimental animals, fasudil protected the liver from ischemia-reperfusion injury by shifting the Kupffer cells/monocytes from M1 to M2 phenotype in the liver and peripheral blood [29]. In *in vitro* cultured macrophages, fasudil can shift macrophages from M1 to M2 phenotype in an experimental autoimmune encephalomyelitis model [15]. However, these studies did not elucidate whether fasudil directly or indirectly regulated macrophage polarization. With regards to the complex natural environment of macrophages in kidney, we speculate that the effect of fasudil on M1/M2 ratio may be multifactorial in DN. Although a recent study has reported that depletion of macrophage improves renal function in DN mice [30], the renal protective effects of fasudil do not only depend on the regulation of macrophage polarization. It has been previously reported that reducing the apoptosis and necrosis of tubules was involved in the renal protective function of fasudil against cisplatin-induced acute kidney injury [31]. It has also been reported that fasudil restored renal function by suppressing renal tubular apoptosis, ameliorating redox imbalance, and DNA damage in contrast-induced acute kidney injury [32], which may be related to its inhibitory effect of the ROCK signaling pathway.

A large number of studies have indicated that the activation of the ROCK signaling pathway might be involved in DN development through the following mechanisms: (1) mediating the activation of NF-*κ*B, fibronectin, and TGF-*β* to accelerate glomerulosclerosis [33]; (2) inhibiting podocyte formation [34] and interfering with contraction movement to increase the glomerular permeability and promote urinary protein formation [35, 36]; (3) mediating epithelial–mesenchymal transition (EMT) to accelerate renal interstitial fibrosis [37–39]; and (4) contracting the renal vascular network, worsening renal hemodynamics, and aggravating kidney damage [40, 41]. In this study, the p-MYPYT1 protein level increased in the mice of the DM group, suggesting ROCK activation in renal tissues of diabetic mice. After fasudil intervention, the p-MYPT1 protein level decreased, suggesting ROCK activity was suppressed. In addition, we showed that the phosphorylation level of p-MYPYT1 was positively correlated with the level of iNOS (an M1-specific maker) and was negatively correlated with the level of Arg-1 (an M2-specific maker), indicating that ROCK activity correlated with macrophage polarization in renal tissues of diabetic mice. Cheng et al. [42] showed that macrophages cultured in a high glucose environment had various biological behaviors including activation of the ROCK signaling pathway and regulation of macrophage adhesion and secretion. Meanwhile, Matoba et al. [43] also showed that TNF-*α* induced MCP-1 expression in glomerular mesangial cells through the ROCK signaling pathway to participate in the glomerular macrophage exudation process. Moreover, it has been reported that fasudil can act on *in vitro* cultured macrophages, shifting macrophages from M1 to M2 phenotype [15]. Therefore, regulation of macrophage polarization by fasudil in DN development might ascribe to inhibiting the ROCK pathway. It should be pointed out that there are limitations in this study. Firstly, these results need to be further confirmed in *in vitro* studies. Secondly, this study focused on the relationship between fasudil and macrophage polarization in DN development. Although we found that fasudil reduced urinary protein and Scr in diabetic mice, the major limitation is a lack of more detailed analysis of kidney injury. However, in support of our findings, it has been reported that fasudil can attenuate kidney injury in the diabetic animal models [44–46]. Future research will focus on the emerging issues from this study and on the detailed molecular mechanisms.

In summary, fasudil can inhibit M1 macrophage polarization and induce M2 macrophage polarization in DN progression. The use of fasudil is expected to slow the progression of diabetic nephropathy. In addition, this study preliminarily explored the association between the ROCK signaling pathway and macrophage polarization in the development of DN. Whether macrophage polarization in DN is regulated by the ROCK signaling pathway still requires further studies.

## Data Availability

The data used to support the findings of this study are included within the article.
